# The T-cell Response to Epstein-Barr Virus–New Tricks From an Old Dog

**DOI:** 10.3389/fimmu.2019.02193

**Published:** 2019-09-18

**Authors:** Heather M. Long, Benjamin J. Meckiff, Graham S. Taylor

**Affiliations:** Institute of Immunology and Immunotherapy, University of Birmingham, Birmingham, United Kingdom

**Keywords:** resident memory, nasopharyngeal carcinoma (NPC), tumor virus, DLBCL - Diffuse large B cell lymphoma, Burkitt lymphoma, gamma delta T cells, cytotoxic CD4 T cell, Hodgkin Lymphoma

## Abstract

Epstein-Barr virus (EBV) infects most people and establishes life-long infection controlled by the host's immune system. The genetic stability of the virus, deep understanding of the viral antigens and immune epitopes recognized by the host's T-cell system and the fact that recent infection can be identified by the development of symptomatic infectious mononucleosis makes EBV a powerful system in which to study human immunology. The association between EBV and multiple cancers also means that the lessons learned have strong translational potential. Increasing evidence of a role for resident memory T-cells and non-conventional γδ T-cells in controlling EBV infection suggests new opportunities for research and means the virus will continue to provide exciting new insights into human biology and immunology into the future.

## Introduction

Epstein-Barr virus (EBV) was first identified in 1964 in a biopsy from a patient with Burkitt Lymphoma (1). This gammaherpesvirus has co-evolved with humans for millennia and is a highly successful pathogen, infecting 90–95% of people worldwide who then carry the virus for life. EBV infection normally occurs in young children with few if any symptoms (2). However, if acquisition is delayed to adolescence then 25–75% of those infected develop infectious mononucleosis (IM). This is an acute syndrome characterized by a tetrad of symptoms: fever, fatigue, sore throat, and lymphadenopathy (3, 4). The acute symptoms of IM usually resolve by themselves, but serious rare complications may occur which include airway obstruction and splenic rupture (2). Longer term, a history of IM is associated with a raised incidence of Hodgkin lymphoma (HL) in the decade following infection and an increased risk of developing multiple sclerosis (5, 6).

EBV transmission occurs orally. Initial infection and replication of the virus most likely occurs in epithelial cells and locally infiltrating B-cells, resulting in high levels of virus shedding in the oropharynx (7–9). This lytic stage of viral replication is driven by up to 80 viral genes expressed in a temporally regulated manner (7). At the same time, the virus drives a proliferation of B-cells by activating its growth transforming programme. Here, viral gene expression comprises six Epstein-Barr Nuclear Antigens (EBNAs: EBNA1, EBNA2, EBNA3A, EBNA3B, EBNA3C, EBNA-LP), two latent membrane proteins (LMPs: LMP1, LMP2) and the viral anti-apoptotic protein BHRF1 (7, 10). This programme leads to the expansion of EBV-infected B cells in the oropharyngeal lymphoid tissue and the appearance of infected B cells in the blood. In some infected B-cells the virus downregulates its growth transforming program allowing the cells to enter the memory B-cell pool with the virus persisting as a truly latent infection lacking viral gene expression (11). These infected B-cells circulate between the blood and oropharyngeal lymphoid tissue and, in the latter, may occasionally switch into lytic cycle, releasing infectious virus for transmission to new hosts and for infection of new local B cells to maintain the viral reservoir (7).

EBV has potent growth transforming activity, as demonstrated *in vitro* by its ability to efficiently transform B-cells into immortalized lymphoblastoid cells lines (LCLs) (12). This property of the virus is used by laboratories worldwide to simply and reliably generate permanently growing B cell lines for research (13). The virus also has oncogenic potential, as demonstrated by its association with several malignancies that together total almost 200,000 cases of cancer each year worldwide (14). Nevertheless, the large majority of people infected by EBV do not suffer any long-term ill effects from the virus. This is due to the anti-viral immune response which, although unable to eliminate the virus, counters primary EBV infection and then maintains subsequent lifelong control to enable mutual co-existence of the virus and its host (8). Early control of EBV infection is associated with expansion of innate immune cells (primarily NK cells, described by Professor Munz in this review series) and of CD8+ and CD4+ T-cells specific for a broad range of EBV proteins expressed during the lytic and latent stages of viral infection (8). Over time, these T-cell responses decrease in magnitude but persist for the life time of the host. Low frequencies of latently EBV-infected B-cells can, nevertheless, be detected in the circulation (15) and infectious virus is periodically produced in the oropharynx and secreted in saliva (16, 17). Therefore, despite the exuberant primary immune response that occurs immediately after infection, and subsequent long-term immune surveillance, the virus is able to successfully persist for life. This apparent détente can, however, be broken if the balance between the virus and its host's immune response is disrupted. The clearest demonstration of this is in immunosuppressed patients, where loss of immune control of EBV can allow virus reactivation and the accumulation of EBV-transformed B cells, leading to post-transplant lymphoproliferative disease (PTLD) (18).

## The EBV-specific T-cell Response During Symptomatic Primary Infection

Most work studying T-cell responses during primary infection has investigated people identified as having been recently infected with EBV through the overt symptoms of IM. The results of such studies are valuable but need to be interpreted with two caveats. First, in contrast to the vast majority of individuals who acquire EBV asymptomatically in early childhood, IM represents an atypical pathological state. Second, viral infection occurs several weeks prior to symptoms developing and samples being taken (19). On presentation, IM patients have unusually high numbers of atypical lymphocytes in the blood, the magnitude of which can resemble leukemia (20). Detailed analysis of blood from these patients shows that the majority of the expanded lymphocytes are EBV-specific T-cells (8). These largely comprise CD8+ T-cells specific for the EBV lytic cycle proteins with a clearly defined hierarchy. Most are specific for immediate early EBV lytic cycle proteins, a smaller number are specific for early proteins with few specific for late proteins (21–24). CD8+ T-cells specific for latent cycle proteins are also expanded but to a smaller degree. Of these, most are specific for the EBNA3A, 3B, and 3C proteins with a lower frequency of LMP2-specific T-cells also present (25, 26). Responses to the EBNA1 protein occur sporadically in IM patients bearing particular HLA alleles, such as HLA-B^*^3501; that are uncommon in the general population. In people with these alleles, however, the EBNA1-specific CD8 T-cell response is strong (27). The phenotype of the CD8+ T-cell response has been explored using HLA-class I tetramers. As might be expected the EBV-specific CD8+ T cells are proliferating and highly activated, expressing HLA-DR, CD38, and CD69 (28). They also express the CD45RO isoform, lack expression of the lymphoid homing markers CCR7 and CD62-L (26, 29), and are highly susceptible to apoptosis, likely due to low expression of the anti-apoptotic protein bcl-2 (30, 31). Given their extreme sensitivity to apoptosis *in vitro*, with significant cell death occurring within just a few hours, measurement of response size using directly *ex vivo* methods such as HLA tetramer staining provide the most accurate enumeration. Studies in IM patients using HLA tetramers report that CD8+ T-cells specific for individual EBV lytic and latent epitopes can account for 1–40 and 0.1–5% of total CD8+ T cells, respectively (25, 26, 28).

Regarding the EBV-specific CD4 T-cell response, during IM weak responses to lytic and latent cycle antigens are present with the former observed more frequently (32, 33). This early research utilized cytokine secretion assays to detect T-cells reactive to recombinant antigens or lysates of EBV-infected cells. As described above, the propensity of EBV-specific T-cells from IM patients to undergo apoptosis *in vitro* may have limited the sensitivity of this work. The recent development of HLA class II tetramers has allowed this obstacle to be overcome and the CD4+ T-cell response has now been accurately measured (34). *Ex vivo* HLA tetramer staining and flow cytometry has revealed that, although the overall size of the CD4+ T cell compartment does not appear to be expanded (3), the early EBV-specific CD4+ T-cell response is much stronger than previously appreciated. Responses to individual epitopes can reach as high as 1.5% of total CD4+ T-cells (34). Unlike the CD8+ T-cell response, for CD4+ T-cells the latent antigen responses numerically dominate the lytic. The exception is EBNA1, where CD4+ T cell responses are undetectable or low in the blood of patients with IM for several months before they develop (34, 35). Interestingly, this delayed appearance is in line with the previously documented but unexplained delay in EBNA1-specific IgG antibodies (36, 37). Akin to the CD8+ T cell response, EBV-specific CD4+ T cells express high levels of CD38 and CD45RO and lack the lymphoid homing markers CCR7 and CD62L (34). Considering the magnitude of the T-cell response to individual antigens, CD4+ T cells are smaller in number than CD8+ T-cells. However, the CD4+ T cell response is broader and targets more epitopes (8, 38–40). The breadth of the CD4+ T-cell response means that the total EBV-specific expansion is substantial and the overall activation status and phenotype of the total CD4+ T cell pool is altered within IM (34).

The functional profile of EBV-specific CD4+ T cells is consistent with Th1-like cells: most express T-bet and the predominant cytokine produced in *ex vivo* stimulation assays is IFNg. Some cells also produce TNFα and/or IL-2, either in combination with IFNg or alone (41, 42). Early observations of raised perforin expression within the total CD4+ T cells of IM patients suggested that cytotoxic CD4+ T cells are present during primary EBV infection (43). Recent *ex vivo* HLA class II tetramer analysis has now shown that in fact the majority of activated EBV-specific CD4+ T cells express both perforin and granzyme B. Importantly, these cytotoxic proteins were not detected in co-existing influenza A-specific memory CD4+ T cells demonstrating that such expression was not due to non-specific bystander activation (42). Testing with EBV peptides has shown that some EBV-specific CD4+ T cells upregulate cell surface CD107a indicating degranulation and release of perforin and granzyme is possible (41). These observations strongly suggest that EBV-specific CD4+ T cells can exert cytotoxic function *in vivo*; if this is the case they could be highly effective against MHC-II positive EBV infected B cells.

Over time the symptoms of IM resolve with a concomitant decrease of both EBV DNA load and the frequency of EBV-specific CD8+ and CD4+ T-cells in the peripheral blood to values typical of life-long virus carriage (23, 34, 44, 45). All specificities decrease in magnitude although the predominant lytic antigen-specific CD8+ T-cell responses decline the most (25, 26, 34). The phenotype of the T-cells also changes. Activation marker expression decreases and anti-apoptotic proteins such as bcl-2 are upregulated (46). Latent antigen-specific CD8+ T-cells also begin to upregulate lymphoid homing markers allowing them to begin entering the tonsil, followed later and to a lesser extent by lytic antigen-specific CD8+ T cells (23).

## The EBV-specific T Cell Response During Asymptomatic Primary Infection

Although the above studies have informed our understanding of primary infection, most EBV infections occur in the absence of IM (2). Identifying newly-infected asymptomatic individuals is extremely challenging but has been achieved by several longitudinal studies that tracked EBV seronegative individuals over time. Together these studies provide an insight into the immunological events occurring in response to EBV infection in the absence of any clinical manifestation of disease. Early studies of newly infected infants showed no perturbations of the lymphoid compartment or febrile illness (47, 48). Newly infected African children had high levels of EBV DNA in the blood but no change in the overall size of the CD8 compartment. High frequencies of activated EBV-specific CD8+ T-cells could nevertheless be detected (49). Similarly, newly infected young adults also had high EBV DNA load in their blood but lacked lymphocytosis and the size of their T-cell compartment was unchanged (50). A recent prospective study of University students in the United Kingdom identified several individuals undergoing silent infection (51). Each had high EBV viral loads in the blood, reminiscent of IM, but no marked disturbance of total T cell or NK cell frequencies. Of three individuals with the highest viral loads, two had concurrent expansions of EBV-specific CD8+ T cells. In the third individual, EBV-specific T cells did not appear in the blood for several months until the peripheral viral load had decreased (51). Taken together, these studies strongly suggest that in asymptomatic individuals EBV infection elicits a virus-specific CD8+ T cell response. Although lower in magnitude than that seen in IM, this response is nevertheless sufficient to control the infection. The overall conclusion from this body of work is that the characteristic symptoms of IM result from the globally large expansions of highly-activated EBV-specific T-cells, which are predominantly CD8+.

## The EBV-specific T-cell Response During Persistent Infection

EBV-specific T-cell responses are readily detected in the blood of healthy EBV carriers and are present at similar frequencies regardless of whether an individual experienced symptomatic or asymptomatic primary infection (8). CD8+ T-cell responses to lytic and latent cycle antigens are present, the former occurring at higher frequency. Individual lytic epitope-specific responses can account for up to 2% of the total CD8+ T-cell population. The lytic antigen hierarchy seen in IM patients is broadly maintained in memory: responses to immediate early antigens dominate those to early antigens and responses to late-expressed antigens are rare (22, 24). For latent antigen-specific responses, CD8+ T-cells targeting the EBNA3A, 3B, and 3C proteins are dominant. Fewer sub-dominant responses specific for EBNA1, EBNA2, and LMP2 are present; responses against EBNA-LP and LMP1 are rare (8). This general rule is, however, not observed in individuals possessing particular HLA types. Thus, individuals carrying HLA-B^*^3801 possess strong responses to an EBNA2 epitope and those carrying HLA-A^*^0203 possess strong responses to an epitope from EBNA-LP (30, 52). Phenotyping of HLA-I class tetramer-stained cells shows that the EBV-specific T-cell repertoire in persistently infected individuals contains resting antigen-experienced T-cells that are neither activated nor proliferating (30, 52). However, upon antigen challenge these cells exhibit potent effector functions including cytotoxicity and cytokine secretion (29, 52). Expression of lymphoid homing markers, such as CCR7 and CD62L, are variable but are generally expressed more frequently on T-cells specific for latent antigen compared to lytic antigens (52). The phenotype, functional profile and TCR clonotype composition of the virus-specific CD8+ T-cells is stable over many years (53, 54).

Compared to the CD8+ T cell response, the EBV-specific CD4+ T-cell response in healthy carriers is much smaller but the greater diversity of epitopes targeted by these cells in IM is maintained (34, 38–40, 55). Considering responses to individual epitopes, the CD4+ T-cell response is often 10-fold lower than the CD8+ T cell response to the same antigen (33, 34, 38, 39, 56). Across different antigens, latent antigen-specific responses outnumber lytic antigen-specific responses in magnitude. Lytic antigen-specific CD4+ T-cells are equally distributed against the immediate early, early and late antigens (39, 40); the heavy skewing exhibited by CD8+ T-cells is absent. HLA class II tetramer analysis shows that EBV-specific memory CD4+ T cells have the same phenotype regardless of whether they target latent or lytic antigens. They do not express activation markers and are evenly distributed between the CCR7+ central memory and CCR7- effector memory subsets (34). Compared to CD4+ T-cells in IM patients, they no longer express perforin and granzyme, and upon *ex vivo* stimulation their cytokine polyfunctionality is increased with TNFa being the most predominant cytokine produced (41, 42, 57).

## Location, Location, Location: The Emerging Importance of Resident T-cell Memory

Much of our understanding of EBV-specific T cell immunity has come from studying circulating virus-specific T cells because sampling blood lymphocytes is convenient and minimally invasive. However, such analyses do not provide a complete picture of overall immune control. Recent studies in mice have highlighted the vital role of local immune responses, including virus-specific tissue resident memory T-cells (Trm cells), in providing long-term protective immunity against viral infection (58). Trm cells reside at sites of infection, where they enable rapid local immune responses against reactivation or secondary infection. Studying immunity in human tissues naturally presents a far greater challenge. Nevertheless, CD8+ and CD4+ Trm cells have been detected in a range of lymphoid and non-lymphoid tissues (59). These cells are transcriptionally distinct from circulating memory T cell populations (60).

Investigation of local EBV-specific T cell responses within tissues has been achieved by analysis of tonsils, which represent one of the major oropharyngeal sites of EBV infection and reactivation. In IM patients EBV loads in the tonsils are high yet EBV-specific CD8+ and CD4+ T cells are markedly lower in frequency at this site than in the blood of the same individual (23, 34, 42). Unlike lymph nodes, where T-cells may enter passively via afferent lymph vessels, entry into tonsils is dependent on transition across high endothelial venules (61). Expression of the lymphoid homing markers required for this process, CCR7 and CD62L, is highly downregulated on EBV-specific T cells in IM patients (26, 28, 34). This likely explains why EBV infection in the oropharynx is inefficiently targeted by virus-specific T cells during IM. As the symptoms of IM resolve, latent antigen-specific CD8+ T cells in the blood re-express CCR7 and CD62L and this coincides with their increasing frequency in the tonsil. At this time, some tonsillar latent antigen-specific CD8+ T cells express high levels of the activation marker CD38, consistent with encountering antigen at that site. In contrast, lytic antigen-specific CD8+ T cells remain CCR7-negative in the blood for several months after IM and their accumulation in the tonsils is correspondingly delayed. This delay may explain why lytic infection is not controlled in the throat of IM patients who continue to shed virus in saliva for many months after primary infection (23, 44).

In long-term EBV carriers the picture is reversed. Latent and lytic antigen-specific CD8+ T cells are higher in frequency in the tonsils than in the blood and the accumulated tonsillar EBV-specific CD8+ T cells no longer express the activation marker CD38. The degree of enrichment in the tonsils varies for latent vs. lytic antigen specific T-cells, with the former preferentially enriched compared to the latter (10-fold and 4-fold enrichment in the tonsil respectively) (23). This variable enrichment reflects the expression of CCR7 and CD62L on T-cells in the blood (26, 28) but other factors are likely involved. Thus, enrichment of EBV-specific CD8+ T-cells occurs only in the tonsils (and presumably other oropharyngeal lymphoid tissues) but not lymph nodes from other anatomical sites nor bone marrow (62, 63). The TCR repertoire of EBV-specific CD8+ T-cells within tonsils and blood of the same individual shows little difference (31) suggesting that tonsillar T-cell enrichment is not the result of selective recruitment or clonal expansion with the tonsil site.

Most EBV-specific CD8+ T cells in the tonsils of long-term EBV carriers express CD69 (64), one of the distinguishing markers of Trm cells (60). CD69 is a C-type lectin that mediates T cell retention in tissues and secondary lymphoid organs through sequestration of sphingosine-1-phosphate receptor (S1PR), a key molecule required for egress (65, 66). Although CD69 is also transiently expressed upon T cell activation (67) there is no concurrent raised expression of other cellular activation markers, including CD38 and HLA-DR, suggesting that CD69 expression in this context reflects active T cell retention rather than activation. In long-term EBV carriers, many tonsillar EBV-specific CD8+ T cells also express CD103 (αEβ7), an integrin that binds to E-cadherin and mediates retention at epithelial sites (23, 64). In contrast, this protein is not detected on the lower frequency EBV-specific CD8+ cells present in the tonsils of IM patients (23). Importantly, the CD103+ CD8+ subset of T cells from long-term EBV carrier tonsils show greater sensitivity *in vitro* to stimulation with cognate antigen (68). Elegant immunofluorescence microscopy of human tonsils has revealed that CD69+CD103+ CD8+ T cells preferentially localize at or near the tonsillar lymphoepithelial barrier (64). Although analysis of antigen specific T cells was not possible in this study, this observation suggests that EBV-specific CD8+ T cells of this phenotype may be retained in close proximity to the tonsillar epithelium, the region of the tonsils where EBV-positive B cells are predominantly found (69, 70).

## Beyond αβ: Accumulating Evidence of a Role for γδ T-cells

All of the research described above has studied the αβ subset of T-cells and how these respond to EBV in primary and persistent infection. These T-cells express T-cell receptors composed of a heterodimeric α and β chain which enables them to recognize peptide epitopes presented by HLA molecules (8). A second subset of T-cells exists that express a different T-cell receptor formed from a heterodimeric γ and δ chain. These γδ T-cells can, in humans, be broadly divided into two groups based on the type of δ chain they express. T-cells expressing the Vδ2 chain are more abundant in the blood, where they comprise 90% of the circulating γδ T-cell pool (71). Cells expressing the Vδ1 chain occur at low levels in the blood but comprise the bulk of γδ T-cells in tissues (72).

There is good evidence from mouse studies that γδ T-cells are involved in protection from herpesviruses such as murine cytomegalovirus (73, 74). A role for γδ T-cells in cancer is also suggested both by studies in mouse cancer models (75) and in humans by associations observed between intratumoural γδ T-cell frequency and prognosis (76). Several lines of evidence now show that γδ T-cells also play an important role in controlling EBV infection and transformation.

## γδ T-cells in Infectious Mononucleosis

Only a small number of studies have examined γδ T-cell during IM. Two studies, each analyzing 10 IM patients and a number of matched controls, showed an increased frequency of γδ T-cells in the blood by flow cytometry, with up to a 4-fold increase in absolute number (77, 78). The majority of γδ T-cells in IM patients were positive for the cell surface activation marker CD38 whereas this marker was absent from γδ T-cells in healthy donors (77) A larger study detected increased αβ and γδ TCR gene expression in the blood of IM patients by transcriptional analysis. Based on a deeper analysis of the transcriptome the authors suggest that both the Vδ1 and Vδ2 subsets are increased in IM (79). Because increases in RNA levels may not necessarily reflect changes in cell frequency the authors performed confirmatory flow cytometry analysis of a subset of patients which demonstrated a 3.4-fold increase in γδ T-cell frequency in IM (79). Note that the antibody used for this work was unable to differentiate between the two γδ T-cell subsets so the relative contribution each makes to the overall expansion of γδ T-cells in IM requires further investigation.

The above observational studies cannot determine whether the increases in γδ frequency that occur in IM are an indirect result of bystander activation or represent direct recognition of EBV infected cells. An experiment of nature suggests the latter may be the case. An EBV-negative recipient of a cord blood transplant, who acquired EBV 31 days after transplantation, experienced prolonged high-level EBV viremia yet did not develop any clinical manifestations of EBV-associated disease. This patient lacked detectable EBV-specific αβ T-cells by HLA class I tetramer staining and interferon-gamma ELISpot assays (80) but had large expansions of γδ T-cells that reached almost 50% of total T-cells. These cells were mostly Vδ1 T-cells but a smaller number of Vδ2 T-cells were also present. The Vδ1 T-cells were predominantly CD45RA- CD27+ central memory cells and, based on their expression of CD57, the authors concluded they were activated. Interestingly, the γδ T-cells were able to degranulate when exposed to an EBV+ve cell line *in vitro* suggesting direct recognition of EBV+ve cells was possible. In a separate study, an abundant Vδ1 T-cell clone isolated from a recipient of an allogeneic stem cell transplant killed autologous and allogeneic LCLs but not the EBV-ve Raji Burkitt Lymphoma (BL) cell line (81).

## γδ T-cells and EBV-associated Neoplasms

EBV is associated with malignancies arising in different cell backgrounds. Examples include BL, a tumor of B cells that occurs predominantly in Sub Saharan Africa, and nasopharyngeal carcinoma (NPC) an epithelial carcinoma that occurs at high incidence throughout South East Asia. γδ T-cells are altered in patients with these EBV+ve malignancies. Two papers studying patients with NPC both report that while the frequency of γδ T-cells in patients is unaltered their functional capacity is impaired. Following *in vitro* culture, peripheral blood mononuclear cells (PBMCs) from NPC patients yielded smaller numbers of γδ cells compared to control donors and were unable to kill an NPC cell line (82). When tested in cytotoxicity assays, γδ T-cells from patients lacked the ability to kill CNE-2, a tumor cell line established from an NPC patient (82). Interestingly, this deficit in NPC cell killing was found only in patients with active NPC since γδ T-cells from successfully-treated NPC patients exhibited the same level of cytotoxicity against CNE2 as those from control donors. Similarly, another research group found that Vγ9Vδ2 T-cells from NPC patients, but not healthy donors, were unable to lyse the NPC cell line HK1 or the control cell line K562 when tested directly *ex vivo* (83). In this study Vγ9Vδ2 T-cells from NPC patients also produced less interferon-gamma and TNFα than cells from control donors when exposed to HMBPP, a phosphoantigen that is a potent stimulator of Vγ9Vδ2 T-cell activity. Flow cytometry analysis showed that Vγ9Vδ2 T-cells in NPC patients were more highly differentiated, with a smaller proportion of central memory and higher proportion of terminally differentiated TEMRA cells. A study in BL patients in Ghana reports that patients have lower Vγ9Vδ2 T-cell frequency than healthy donors (84). Whether the functional capacity of Vγ9Vδ2 T-cell in BL patients is impaired is unknown.

In several of the above papers, γδ T-cells were reported to be capable of recognizing EBV+ LCLs (80–82), the NPC cell line HK1 (83) or the NPC cell line CNE2 (82); note that experiments using CNE2 need cautious interpretation as it has been shown to be contaminated with HeLa (85). These observations raise the question of which cellular targets render EBV+ve cells visible to γδ T-cells. In this regard it is important to point out that γδ T-cell recognition of target cells is complex and can involve the T-cell receptor and/or ligands for natural killer receptors they express, such as NKG2D (86, 87).

## γδ T-cell Recognition of EBV+ve Cells

The BL line Daudi (88) is a reliable stimulator of Vγ9Vδ2 T-cells and has been used for many years for this purpose (89–99). These studies also often included other BL cell lines which in contrast showed lower or indeed no γδ T-cell stimulatory activity. Indeed, the BL line Raji, as well as LCLs, were often used as a negative controls in such work. It is now known that Daudi overproduces endogenous non-peptidic phosphorylated metabolites due to upregulation of the mevalonate pathway (96) resulting in intracellular accumulation of IPP, a host phosphoantigen counterpart of HMBPP (100). Vγ9Vδ2 T-cell phosphoantigen sensing is dependent on: (i) target cell exposure to/accumulation of HMBPP and IPP and (ii) target cell expression of BTN3A1, which binds phosphoantigens via its intracellular B30.2 domain (101). It is currently unclear why most BL cell lines are unable to generate Vγ9Vδ2 T-cell lines when co-cultured with PBMCs, however, this potentially could reflect deficient expression of BTN3A1 or insufficient IPP production. These possibilities require further investigation.

Interestingly, being able to generate Vγ9Vδ2 T-cell lines and being able to be recognized by those same cells appear to be distinct properties. Thus, Daudi-stimulated Vγ9Vδ2 T-cells are able to recognize and kill multiple BL lines that themselves lack stimulatory activity for Vγ9Vδ2 T-cells (94). This difference between the ability to generate Vγ9Vδ2 T-cells and being sensitive to γδ T-cell recognition could be due to: (i) activated Vγ9Vδ2 T-cells possessing increased sensitivity to a lower level of phosphoantigens and/or BTN3A1 (ii) Daudi stimulated Vγ9Vδ2 T-cells being able to utilize alternative target recognition mechanisms that are independent of phosphoantigens. For example, Vγ9Vδ2 T-cells expressing NKg2D can recognize target cells expressing ULBP4, a protein upregulated upon EBV infection of B-cells (102).

Additional complexity is suggested by a recent report of a fundamental population-level difference in the response to BL lines (99). After co-culturing PBMCs from 24 donors with EBV+ve Akata BL cells for 10 days *in vitro*, Djaoud and colleagues found that 13 co-cultures had large expansions of Vγ9Vδ2 T-cells and NK cells. In contrast, the other 11 co-cultures had a large expansion of NK cells but only very small increases in Vγ9Vδ2 T-cells. Vγ9Vδ2 T-cell expansion was sensitive to an anti-BTN3A1 blocking antibody or mevastatin, an inhibitor of the mevalonate pathway that generates the intracellular phosphoantigens recognized by Vg9Vd2 T-cells. Interestingly, the authors reported that Vγ9Vδ2 T-cell expansion did not occur when the EBV-ve derivative of the Akata BL cell line was used instead. The authors extended their work to include a larger range of EBV+ve stimulator cells. These experiments used the EBV+ve Akata BL cell line as well as the EBV+ve BL cell lines Daudi and Kem-I (all three are type I BL lines expressing EBNA1), Sal (a Wp-restricted BL line that expresses EBNA1 but also EBNAs 3A, 3B, and 3C along with a truncated form of EBNA-LP) and Raji and Jijoye (the EBV+ve variants of these two lines express all EBV latent cycle proteins). Only the type I BL cells (Akata, Daudi, and Kem-I) stimulated Vγ9Vδ2 T-cell expansion (99). This result led the authors to conclude that Vγ9Vδ2 T-cell expansion was a property of type-I BL cells only. Independent confirmation of these results and testing of a larger range of B-cell lines is now needed to determine if Vγ9Vδ2 T-cell recognition is indeed limited solely to type I BL lines and to reveal the underpinning biological mechanisms responsible.

## γδ T-cells in Mouse Models of EBV

Further support for a role of Vγ9Vδ2 T-cells in the control of EBV comes from studies in mice. Growth of CNE2 cells injected subcutaneously into nude mice was slowed following intravenous administration of Vγ9Vδ2 T-cells (103). More advanced mouse models now exist including mice reconstituted with human immune components which, arguably, are better models in which to study EBV immunology (104). Work using an early form of this model, CB.17^scid/scid^ mice [severe combined immunodeficient (SCID) mice] injected intraperitoneally with peripheral blood lymphocytes, showed increases in γδ T-cells following administration of irradiated Daudi cells. These mice were protected against subsequent challenge with non-irradiated Daudi cells but not non-irradiated Raji BL cells which developed into disseminated lymphoma (105).

Control of EBV-driven lymphoproliferations and lymphomas has been demonstrated by two studies that used more advanced mouse models. The first used EBV-transformed LCLs as targets of Vγ9Vδ2T-cells (106). In preliminary *in vitro* work, phosphoantigen-specific Vγ9Vδ2T-cells were unable to recognize LCLs, a result consistent with previous publications. However, when the same Vγ9Vδ2 T-cells were positively selected using magnetic beads they then recognized and killed LCL cells. The authors suggested that binding of the anti-γδ antibody-loaded beads to the Vγ9Vδ2 T-cell receptor may have activated the cells thereby allowing them to recognize LCLs. Cytotoxicity of the T-cells was inhibited by concanamycin A, a widely-used inhibitor of perforin-mediated cytotoxicity. A somewhat surprising observation, however, was that purified bcl-2 protein also inhibited cytotoxicity: how an extracellular protein was able to enter the LCLs to inhibit intracellular granzyme activity was not discussed.

Initial *in vivo* experiments, using immunodeficient Rag2^−/−^ γc^−/−^ mice injected with LCLs, then showed that adoptive transfer of magnetically enriched Vγ9Vδ2 T-cells could prevent LCL-induced lymphoproliferative disease and eliminated established LCL tumors (106). Extending the work to Rag2^−/−^ γc^−/−^ mice carrying human immune components (established by injection of PBMC) showed these mice carried human Vγ9Vδ2 T–cells and that LCL tumors were rejected after the mice were treated with pamidronate, a drug that stimulates Vγ9Vδ2 T-cells (106). The reasons why Vγ9Vδ2 T-cells in these mice did not require activation via bead selection in order to recognize and kill LCLs was not explored. Pamidronate-induced tumor rejection was, however, clearly Vγ9Vδ2 T-cell dependent. These results raise the interesting possibility that patients with PTLD could one day be treated using small molecular agents to activate their own Vγ9Vδ2 T-cells. Several ways to achieve this in the clinic can be envisaged (107) including using zoledronate, a drug licensed to treat bone diseases but which also exhibits potent Vγ9Vδ2 stimulatory activity (96).

Work by another group also suggests that Vγ9Vδ2 T-cells may be able to eliminate LCLs *in vivo* without requiring activation via bead selection. Here the model used was the Rag2^−/−^ γc^−/−^ system but now using human cord blood to reconstituted the mice with human immune components (108). Spontaneous EBV-driven lymphoproliferations could be induced in the mice by infecting the cord blood with EBV immediately prior to injection. Adoptive transfer of Vγ9Vδ2 T-cells (generated *in vitro* but without magnetic bead selection) soon after reconstitution prevented lymphoma development. Delaying adoptive transfer of Vγ9Vδ2 T-cells until tumors were evident retarded tumor growth but did not eliminate them.

Finally, although far fewer studies have investigated Vδ1 T-cell recognition of EBV targets, the available data clearly shows marked differences exists between the Vδ1 and Vδ2 T-cell subsets. Thus, Vδ1 T-cells are not stimulated by Daudi BL cells but are instead stimulated by LCLs (109–111). Regarding other BL lines, in one study, Daudi, Raji, Ramos, BL41, and BL57 did not stimulate Vδ1 T-cells (111) whereas Raji was reported to be stimulatory by another (109). These disparate results may reflect the fact that Vδ1 cells employ a range of target recognition mechanisms (72).

Collectively the above results clearly show that both γδ T-cell subsets warrant far greater attention then has been the case in the past. Which subset will prove to be more important in controlling EBV remains to be determined. Indeed, the critical effector subset may vary as individuals progress from primary infection into the long term carrier state and, for a minority, develop an EBV+ cancer.

## A Role for Natural Killer T (NKT) Cells in Controlling EBV?

A limited number of studies have examined the contribution of NKT cells to EBV immunity. NKT cells are a conserved population of innate-like T cells that express the semi-invariant Vα24-Jα18/Vβ11 T cell receptor. Unlike conventional αβ T cells, NKT cells recognize glycolipid antigens presented by the non-polymorphic MHC class I-like molecule, CD1d (112). Only one study has assessed the frequency of NKT cells in the blood during EBV infection (113). Using flow cytometry, no expansion of CD3+CD56+CD244+ NKT cells was detected in 11 acute IM patients compared to age-matched healthy carriers. A larger study to assess the frequency and function of Vα24-Jα18/Vβ11+ NKT cells is required to confirm this observation. Nevertheless, a possible role for NKT cells in immune control of EBV is supported by the following lines of evidence. (i) NKT cells are significantly lower in frequency in the blood of EBV+ HL and NPC patients than in healthy carriers (114). Furthermore, NKT cells derived from these patients were less functional upon stimulation with the synthetic glycolipid α-GalCer *in vitro*. (ii) In patients with primary immunodeficiency, individuals with genetic mutations that affect the NKT cell lineage are predisposed to develop EBV-associated disease (reviewed by Professor Munz in this series). Importantly, however, such mutations rarely affect NKT cells in isolation and most patients have coexisting defects in NK and/or T cell development and/or function. Therefore, deciphering the precise contribution of NKT cell deficiency to the lack of overall immune control of EBV in these patients is extremely challenging (115). (iii) The presence of NKT cells reduces EBV transformation of B cells *in vitro*. Upon infection of PBMCs with EBV, prior depletion of NKT cells led to both increased numbers of EBV-infected B cells and raised overall viral titers in the cultures (116). Interestingly CD1d expression was lost from the B cell surface during transformation, and NKT cells were subsequently unable to recognize fully transformed LCL cell lines. The authors therefore suggest that NKT cells may be important for early immune recognition of newly EBV-infected B cells, and exert their function prior to EBV-driven downregulation of CD1d (116). Note that similar downregulation of CD1d has been reported in KSHV and HSV-1 infected cells (117, 118), suggesting that evasion of NKT cell surveillance may be a common strategy of herpesviruses.

## Conclusions

Research on EBV continues to reveal new tricks employed by the human T cell immune system to control infection with this ancient virus. Novel techniques have enabled deeper understanding of the targets, function and evolution of the CD8+ and CD4+ T cell responses, and the potential contributions of previously neglected unconventional T cell subsets are increasingly coming to light. Although it is challenging to obtain tissue samples from people, studying EBV-specific immunity at the site of infection is vital if we are to fully understand the interplay between the virus and its host. With this in mind, we note the current dearth of information on intra-tumoural EBV-specific immunity and highlight this area as a priority for future research.

## Author Contributions

All authors listed have made a substantial, direct and intellectual contribution to the work, and approved it for publication.

### Conflict of Interest Statement

The authors declare that the research was conducted in the absence of any commercial or financial relationships that could be construed as a potential conflict of interest.
